# Supplementary material for: Helical ordering of envelope‐associated proteins and glycoproteins in respiratory syncytial virus
Source: EMBO J. 2021 Dec 22;41(3):e109728. doi: 10.15252/embj.2021109728 (PMC8804925; doi:10.15252/embj.2021109728)
Supplement: Supplementary file 1 — Expanded View Figures PDF [file EMBJ-41-e109728-s001.pdf]

Expanded View Figures

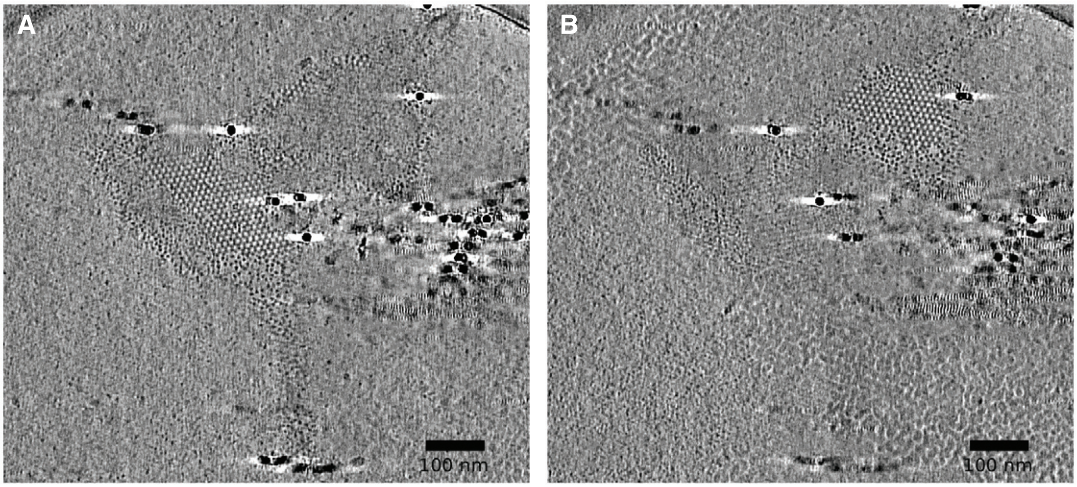

Figure EV1. Honeycomb lattice observed in RSV particles produced in a co-infection study.

A, B Tomograms of a pleomorphic particle show extensive patches of glycoprotein arrays with hexagonal packing. Slices are shown at different *z* values through the same particle.

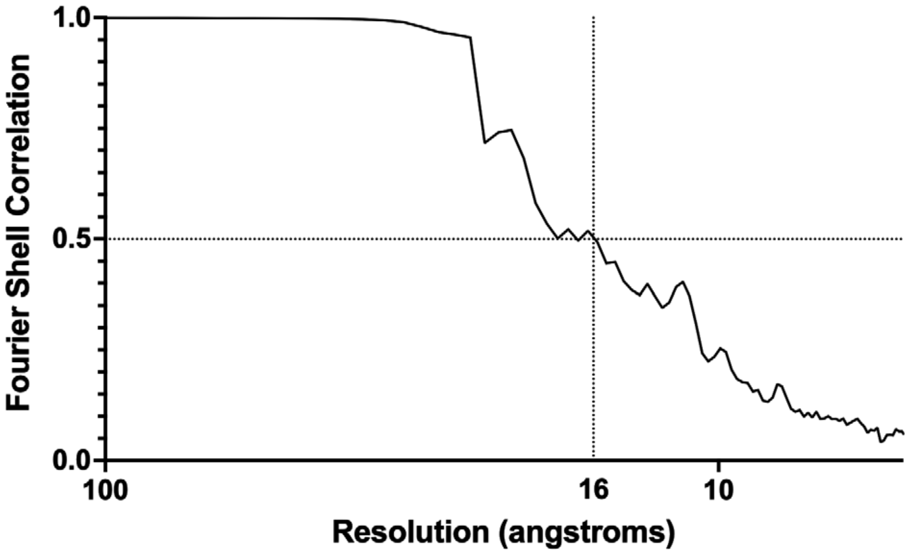

Figure EV2. Resolution measurement for the sub-tomogram average of the RSV viral envelope.

Fourier shell correlation analysis showed that sub-tomogram averaging, focussed on the matrix array, achieved a resolution of 16 Å. Resolution assessment used a cut-off of 0.5, as the gold-standard protocol was not used during sub-tomogram alignment.

 © 2021 The Authors

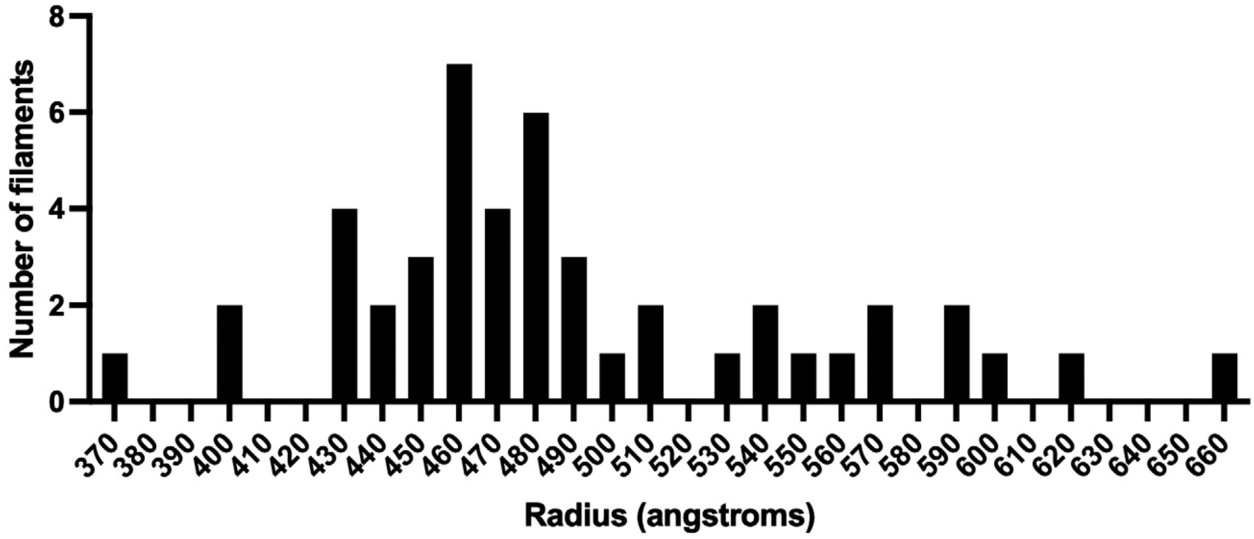

Figure EV3. Radius measurements for the matrix layers in tomograms of RSV virions.

Filamentous virions show a considerable variation in radius. M-layer radii ranged between 370 and 660 Å and appear to show a multi-modal distribution.

© 2021 The Authors

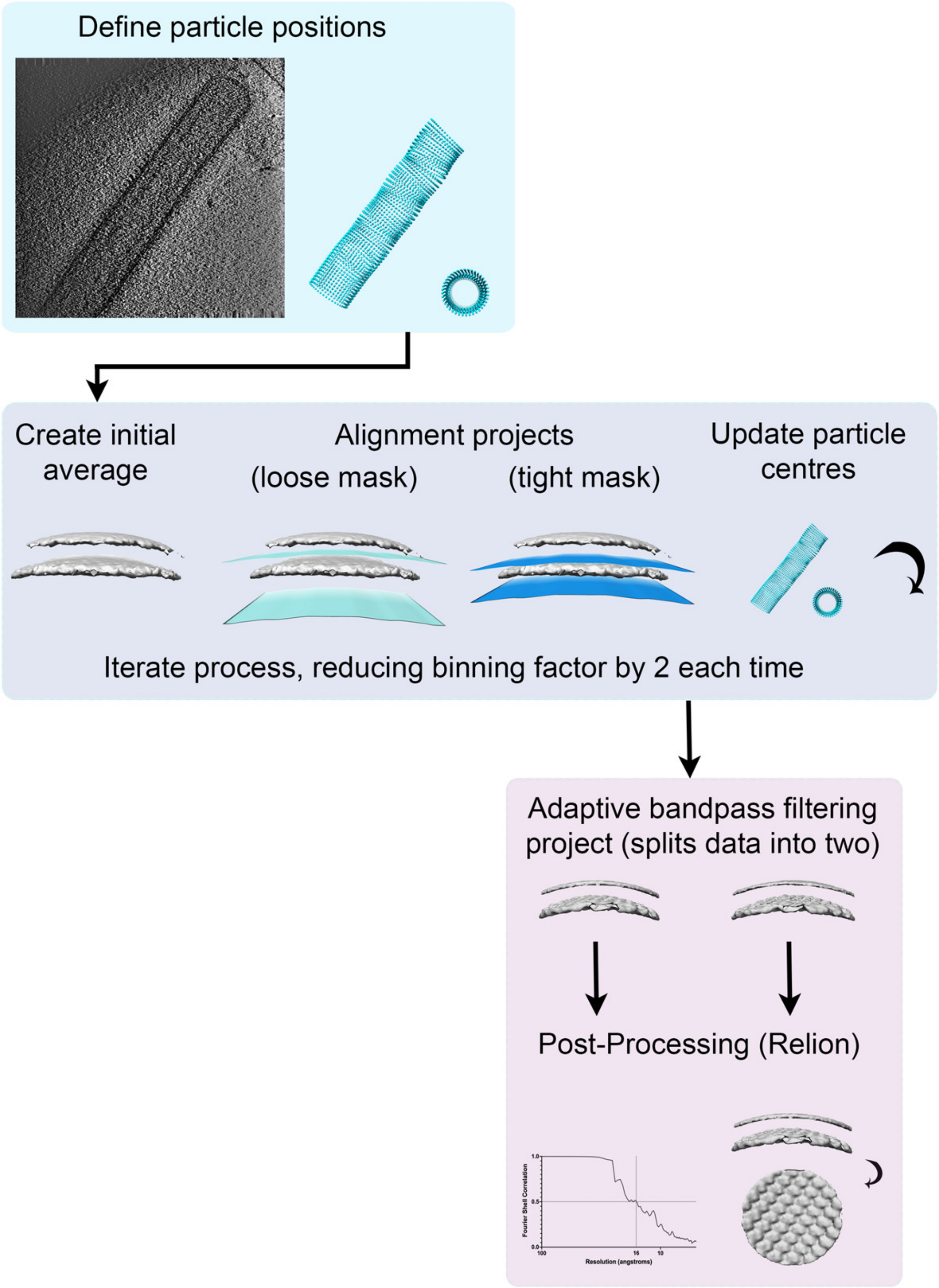

Figure EV4. Schematic diagram of the sub-tomogram averaging process.

Sub-tomogram positions were defined, and alignments were performed using Dynamo. Resolution assessment was performed using Relion.

© 2021 The Authors
