# Supplementary material for: Helical ordering of envelope‐associated proteins and glycoproteins in respiratory syncytial virus
Source: EMBO J. 2021 Dec 22;41(3):e109728. doi: 10.15252/embj.2021109728 (PMC8804925; doi:10.15252/embj.2021109728)
Supplement: Supplementary file 4 [file EMBJ-41-e109728-s004.pdf]

THE EMBO JOURNAL

Helical Ordering of Envelope Associated Proteins and Glycoproteins in Respiratory Syncytial Virus

Michaela Conley, Judith Short, Andrew Burns, James Streetley, Joshua Hutchings, Saskia Bakker, Bridget Power, Hussain Jaffery, Joanne Haney, Giulia Zanetti, Pablo Murcia, Murray Stewart, Rachel Fearns, Swetha Vijayakrishnan, and David Bhella
DOI: 10.15252/embj.2021109728

Corresponding author(s): David Bhella (David.Bhella@glasgow.ac.uk) , Swetha Vijayakrishnan (Swetha.Vijayakrishnan@glasgow.ac.uk)

Review Timeline:

Submission Date:	15th Sep 21
Editorial Decision:	29th Oct 21
Revision Received:	8th Nov 21
Editorial Decision:	30th Nov 21
Revision Received:	1st Dec 21
Accepted:	3rd Dec 21

Editor: Karin Dumstrei

Transaction Report:

(Note: With the exception of the correction of typographical or spelling errors that could be a source of ambiguity, letters and reports are not edited. Depending on transfer agreements, referee reports obtained elsewhere may or may not be included in this compilation. Referee reports are anonymous unless the Referee chooses to sign their reports.)

Dear David,

Thank you for submitting your manuscript to The EMBO Journal. I have now received the reports from two referees. I have been waiting for the third referee to get back, but as I haven't heard anything back despite several emails I don't think that I will receive the third report at this stage. I will therefore move ahead based upon the two reports on hand.

As you can see below, the referees find the analysis interesting. They raise a number of concerns that I presume you will be able to address. I am happy to discuss the revisions further via email or video call if that is helpful.

When preparing your letter of response to the referees' comments, please bear in mind that this will form part of the Review Process File, and will therefore be available online to the community. For more details on our Transparent Editorial Process, please visit our website: https://www.embopress.org/page/journal/14602075/authorguide#transparentprocess

Thank you for the opportunity to consider your work for publication.

I have attached a document with helpful tips on how to prepare the revised version

with best wishes

Karin

Karin Dumstrei, PhD
Senior Editor
The EMBO Journal

Guide For Authors: https://www.embopress.org/page/journal/14602075/authorguide

Revision to The EMBO Journal should be submitted online within 90 days, unless an extension has been requested and approved by the editor; please click on the link below to submit the revision online before 27th Jan 2022:

https://emboj.msubmit.net/cgi-bin/main.plex

Referee #1:

In this study Conley et al. use cryoET combined with subtomogram averaging to describe the organisation of the outer (F and G) and inner (M and M2-1) lattices in RSV virions propagated directly in cryoEM grids, thus avoiding artefacts arising from the purification of the particles, which are very fragile. In general, this is a descriptive work in which the authors note two interesting details that may have biological implications. They observed that the contacts that propagate the lattice of M-dimers in the virus are similar to those observed between dimers in the monoclinic crystals of M grown at neutral pH and that the organisation of the matrix and the glycoprotein lattices are somehow connected and that glycoproteins tend to cluster in pairs. In general terms I would appreciate if the authors had gone a step further and tried to find out whether the glycoprotein dimers they observe are homodimers of F, G, or heterodimers (maybe using FRET), or to try to understand the nature of the connection between the glycoproteins and M. However, I understand that these experiments go beyond the scope of the present work. However, I would appreciate a little more clarity in the text. Here some points I think would be helpful.

MAJOR POINTS

1- The authors mention that they have propagated the virus in three different cell types: vero, U-2 OS and A549. All of them generate filamentous virions with diameters between 90 and 160 nm. Could the authors do a statistical analysis (mean and std of diameter) of virions propagated in different cell lines to see that there is no difference between them? More importantly, it is not clear in which cells did they propagate the virions they are analysing by cryoET. The authors should specify this. If they observe statistical differences between virions propagated in different cells, I think it would be a good idea to study how the matrix is organised in each of them.

2- The observation of the dimers of glycoproteins is one of the strong points of this article, but it would be helpful to understand what their composition is. Why the authors do not obtain a low-resolution reconstruction of these dimers ? Maybe they don't have enough particles ? the authors should discuss whether they have tried.

3- Finally, I find the description of the sub-tomogram averaging a bit confusing. The authors have cropped sub-tomograms of 32

voxels (32x1,79x8=460 A3). A dimer of M is about 60 A in the longest direction. How many dimers of M do they average ? Do they use any kind of symmetry in the process? A supplementary figure illustrating in detail the whole image processing process would help a lot.

MINOR POINTS

1- The authors should introduce better the M2-1 protein in the introduction.

2- There is no panel D in Figure 1.

Referee #3:

Many enveloped viruses are pleomorphic. Some of these viruses, like influenza virus or the respiratory syncytial virus (RSV), can adopt conformations that range from spherical to filamentous; however, in many instances lab-produced virion preparations result in an enrichment of spherical viruses, and a reduction of filamentous viruses. Furthermore, it has been shown that filamentous and spherical viruses have different properties; for example, influenza viruses are mostly filamentous in patients, but they tend to be spherical in laboratory adapted strains.
In this manuscript Conley et al. image infectious RSV virions budding from infected cells, allowing them to characterise filamentous virions. As such, this study is significantly novel compared to others that have described purified RSV virions by cryo-electron microscopy, and can help underpin the understanding of RSV assembly or entry, and potentially the design of antivirals or vaccines.

While the manuscript is very interesting and well written, and most of the conclusions are well supported by the presented results, a number of concerns should be addressed:
- The Fourier transform that the authors present does not convincingly show that the RSV virion in Fig 1B is helical. The authors should clearly acknowledge this in the results section. Additionally, the authors should only show the indexing for half of the Fourier transform, so the reader can assess for themselves the accuracy of the indexing in the other half. The authors should also show the result of filtering the transform on different layer lines and the equator, as was done for influenza virus (PMID: 20498070). Finally, statements that suggest the Fourier transform result is unequivocally helical should be removed, as this is not the case (e.g. "Fourier analysis shows that filamentous particles exhibit helical symmetry" from the introduction).
- The fitting of the M X-ray structure into the subtomogram averaging does not result in an unequivocal orientation. I have tried fitting both a single dimer and a layer of dimers (as the ones shown in movie S2 timepoint 1m 32s), and both orientations result in a reasonably good fit. Do the authors have any further experimental evidence to confirm the orientation they propose is the correct one? Otherwise, they should explain in more detail why they decided one orientation over the other one.
- The description of the "large membranous structure, that might be interpreted as a pleomorphic virion or cell debris" should be completely removed (from text, figures and video). According to the text, it seems this type of structure, and the associated honeycomb lattice, was only observed once. Furthermore, it is not clear if it is a virus or debris. So no conclusions can be made out of it.
- The finding that glycoproteins cluster in pairs is really interesting. However, in the absence of any subtomogram averaging this observation cannot be interpreted. Performing the averaging suggested by the authors in the discussion would greatly improve the significance of the manuscript.

Other minor comments:
- Figure 1 does not have a panel D, but the legend describes it.
- Page 3, "Tomographic reconstruction of filament sections reveal that they are well-ordered." should be "Tomographic reconstruction of filament sections reveal that virions are well-ordered."

Response to reviewers' comments:

Referee #1:

In this study Conley et al. use cryoET combined with subtomogram averaging to describe the organisation of the outer (F and G) and inner (M and M2-1) lattices in RSV virions propagated directly in cryoEM grids, thus avoiding artefacts arising from the purification of the particles, which are very fragile. In general, this is a descriptive work in which the authors note two interesting details that may have biological implications. They observed that the contacts that propagate the lattice of M-dimers in the virus are similar to those observed between dimers in the monoclinic crystals of M grown at neutral pH and that the organisation of the matrix and the glycoprotein lattices are somehow connected and that glycoproteins tend to cluster in pairs. In general terms I would appreciate if the authors had gone a step further and tried to find out whether the glycoprotein dimers they observe are homodimers of F, G, or heterodimers (maybe using FRET), or to try to understand the nature of the connection between the glycoproteins and M. However, I understand that these experiments go beyond the scope of the present work. However, I would appreciate a little more clarity in the text. Here some points I think would be helpful.

We thank the reviewer for their supportive remarks. While we appreciate that readers would, like us wish to learn more about the interactions driving the formation of glycoprotein doublets as well as their interaction with M, we are not able to provide such data without significantly delaying the publication of the present study. Not least, we will likely require additional data to support calculation of a high-resolution structure of the doublet assembly.

MAJOR POINTS

1- The authors mention that they have propagated the virus in three different cell types: vero, U-2 OS and A549. All of them generate filamentous virions with diameters between 90 and 160 nm. Could the authors do a statistical analysis (mean and std of diameter) of virions propagated in different cell lines to see that there is no difference between them? More importantly, it is not clear in which cells did they propagate the virions they are analysing by cryoET. The authors should specify this. If they observe statistical differences between virions propagated in different cells, I think it would be a good idea to study how the matrix is organised in each of them.

We apologise for neglecting to specify which cells were used for each experiment. Text has been added at line 159 and at line 187 to clarify that the cryo-EM experiment with Fourier analysis was performed on RSV propagated in U2-OS cells while cryo-ET was performed using A549 cells. Based on these data we can assess the distribution of particle diameters in these two cell-lines. Measurements taken from filament images at the outer leaflet of the envelope gave mean diameters of 124 nm (SD=27.1, n=30) for virions grown in U2-OS cells and 115.8 nm (SD=12.24, n=47) for

A549 grown virions. Student's t-test indicated that these data were not significantly different ($p=0.1257$).

2- The observation of the dimers of glycoproteins is one of the strong points of this article, but it would be helpful to understand what their composition is. Why did the authors not obtain a low-resolution reconstruction of these dimers? Maybe they don't have enough particles? the authors should discuss whether they have tried.

The reasons we do not have such data to hand are rather mundane and do not warrant discussion in the manuscript, but I am happy to disclose here. Briefly, we did not become aware of the doublet formation phenomenon until after we applied TOPAZ denoising to the data. This approach to denoising tomograms is comparatively recent and was applied as we began to prepare our data for publication. At an earlier stage, we did attempt to produce an average of the glycoprotein spikes. In so doing, the reconstruction appeared to grow additional density on one side – with hindsight, this was likely a consequence of doublet formation. The maps were rather low-quality however and at that point we turned our attention to the matrix layer, which was our primary target. Considering our subsequent discovery, it is unfortunate we did not continue with that line of enquiry.

3- Finally, I find the description of the sub-tomogram averaging a bit confusing. The authors have cropped sub-tomograms of 32 voxels (32x1,79x8=460 A3). A dimer of M is about 60 A in the longest direction. How many dimers of M do they average? Do they use any kind of symmetry in the process? A supplementary figure illustrating in detail the whole image processing process would help a lot.

As can be seen in figure 5 the sub-tomogram average covers ~58 M-dimers. No symmetry is imposed within the volume. At 8× binning the sub-tomogram box is 32×32×32 voxels corresponding to a volume of 32,768 voxels, each of 2,936.5$Å^3$ $((1.79\times8)^3)$ thus the volume of each sub-tomogram is 96,223,021$Å^3$, not 460$Å^3$. Text has been amended at line 538 to make this clearer.

A supplementary figure has been added to support our description of the reconstruction process (figure EV3).

MINOR POINTS

1- The authors should introduce better the M2-1 protein in the introduction.

Additional text has been added at line 101.

2- There is no panel D in Figure 1.

Apologies, the legend for figure D was included in error. The additional figure panel had been included in an earlier draft. As Reviewer #3 has requested inclusion of these data, we have now reinstated the figure panel.

Referee #3:

Many enveloped viruses are pleomorphic. Some of these viruses, like influenza virus or the respiratory syncytial virus (RSV), can adopt conformations that range from spherical to filamentous; however, in many instances lab-produced virion preparations result in an enrichment of spherical viruses, and a reduction of filamentous viruses. Furthermore, it has been shown that filamentous and spherical viruses have different properties; for example, influenza viruses are mostly filamentous in patients, but they tend to be spherical in laboratory adapted strains.
In this manuscript Conley et al. image infectious RSV virions budding from infected cells, allowing them to characterise filamentous virions. As such, this study is significantly novel compared to others that have described purified RSV virions by cryo-electron microscopy and can help underpin the understanding of RSV assembly or entry, and potentially the design of antivirals or vaccines.

We thank the reviewer for their time and supportive comments, it is gratifying that the novelty and importance of our study has been recognised.

While the manuscript is very interesting and well written, and most of the conclusions are well supported by the presented results, a number of concerns should be addressed:

- The Fourier transform that the authors present does not convincingly show that the RSV virion in Fig 1B is helical. The authors should clearly acknowledge this in the results section. Additionally, the authors should only show the indexing for half of the Fourier transform, so the reader can assess for themselves the accuracy of the indexing in the other half. The authors should also show the result of filtering the transform on different layer lines and the equator, as was done for influenza virus (PMID: 20498070). Finally, statements that suggest the Fourier transform result is unequivocally helical should be removed, as this is not the case (e.g. "Fourier analysis shows that filamentous particles exhibit helical symmetry" from the introduction).

We have revised figure 1 accordingly, now the indexing of the lattice is shown over one half of the Fourier Transform, providing readers with a clearer view of the layer lines in the other half. There are

clear layer lines in the Fourier transform, indicative of a helical lattice. Now that we have removed the lattice annotation in figure 1C this is more readily observed. The addition of a filtered image also shows long tracks of density consistent with helical ordering, furthermore the strongest density corresponds to the inner-most of the three layers in the viral envelope – the matrix layer. Text has been revised to use the term helical ordering rather than helical symmetry, thus toning down the assertion that the matrix-layer is a single uninterrupted helical assembly.

- The fitting of the M X-ray structure into the subtomogram averaging does not result in an unequivocal orientation. I have tried fitting both a single dimer and a layer of dimers (as the ones shown in movie S2 timepoint 1m 32s), and both orientations result in a reasonably good fit. Do the authors have any further experimental evidence to confirm the orientation they propose is the correct one? Otherwise, they should explain in more detail why they decided one orientation over the other one.

Forster *et al.* (2015) have established that the M-protein forms stable dimers in solution. Although the sub-tomogram average reconstruction is not sufficiently detailed to show secondary structure elements that would allow us to establish orientations unequivocally, the very close similarity between the lattice seen in the tomograms and the lattice plane in the *C2* crystals investigated by X-ray crystallography indicates strongly that the arrangement we present is the correct one. Our interpretation is supported by the distinctive "C" shape of the M-protein dimers seen in a view perpendicular to the plane of the sheet in Fig. 7A. The model we have presented is the result of applying the 'fit in map' algorithm within UCSF ChimeraX using the coordinates of a single M-dimer, each dimer in the lattice was docked independently. Rotating the coordinates of an M-dimer about the z-axis (in the lattice plane) to any alternate orientation results in significant proportions of the model extending beyond the isosurface contour. Moreover, repeating the fitting calculation returns the model to the orientation we have presented. An alternate interpretation might be postulated where the dimer is rotated about y and translated, such that the negatively charged surface now faces towards the inner leaflet of the envelope (Figure R1). This would seem a less likely scenario given that phospholipid membrane surfaces are negatively charged. Nonetheless, in this orientation, the fit that yields the highest correlation and overlap exhibits severe steric collision with neighbouring dimers while substantial density is not accounted for by the model (alternate fit 1). Measures of overlap and correlation are also poorer in this model. A second fit is possible that superficially, more closely resembles the lattice plane in the published X-ray structure (alternate fit 2), however this model also has multiple clashes and poorer overlap/correlation. Upon close inspection, this model does not preserve the lattice contacts of the published X-ray data. Finally, docking the atomic coordinates in this arrangement requires very precise placement of dimers to prevent jumping to alternate fit 1 when applying the fit in map routine.

In summary, the presented fit is optimal with respect to correlation, overlap, clashes and lattice contacts. Moreover, the fit has been performed independently at various times by three investigators (Conley, Stewart and Bhella) all of whom arrived at the same conclusion.

Orientation	Atoms outside of contour	Correlation at 16Å resolution
Original	499	0.9
Alternate fit 1	580	0.87
Alternate fit 2	727	0.84

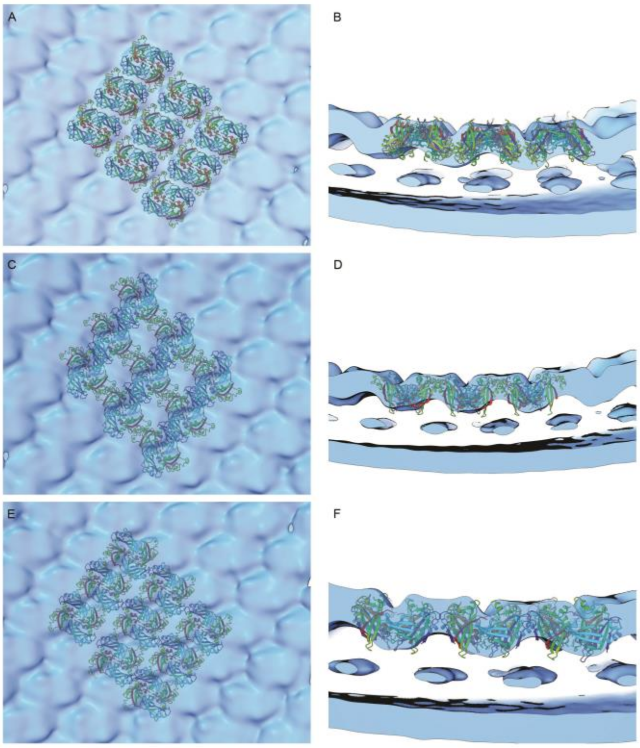

Figure R1 *– Fitting the coordinates of M-dimers to the sub-tomogram average in alternate orientations yields poorer models that exhibit severe steric collision and lower overlap and correlation values.* The original fit is shown (A, B). Alternate fit 1 exhibits severe steric collision (C, D). Alternate fit 2 has fewer severe clashes but also matches the density less well having poorer overlap and correlation than both the original fit and alternative fit 1 (E, F). Neither of the alternative fits preserve the lattice contacts from X-ray crystallography studies.

- The description of the "large membranous structure, that might be interpreted as a pleomorphic virion or cell debris" should be completely removed (from text, figures and video). According to the text, it seems this type of structure, and the associated honeycomb lattice, was only observed once. Furthermore, it is not clear if it is a virus or debris. So no conclusions can be made out of it.

While it is the case that this feature is infrequently seen, our later studies specifically targeted filamentous rather than pleomorphic virions. We have however observed this phenomenon both prior to this study and since. We first noted hexagonal surface features in negative stain images of purified RSV virions recorded many years ago. These images were not published and are assumed to now

be in the possession of Prof. R.J. Sugrue (Nangyang Technical University, Singapore). More recently in a study of chimeric virions that is currently under review elsewhere (https://doi.org/10.1101/2021.08.16.456460) we noted the presence of extensive hexagonal arrays of glycoproteins (Figure R2). The data were not included in that article however and the complexity of that study makes inclusion of those data in this paper undesirable. Based on these unreported findings, we consider that this is an authentic and potentially important observation and have retained it in this revision.

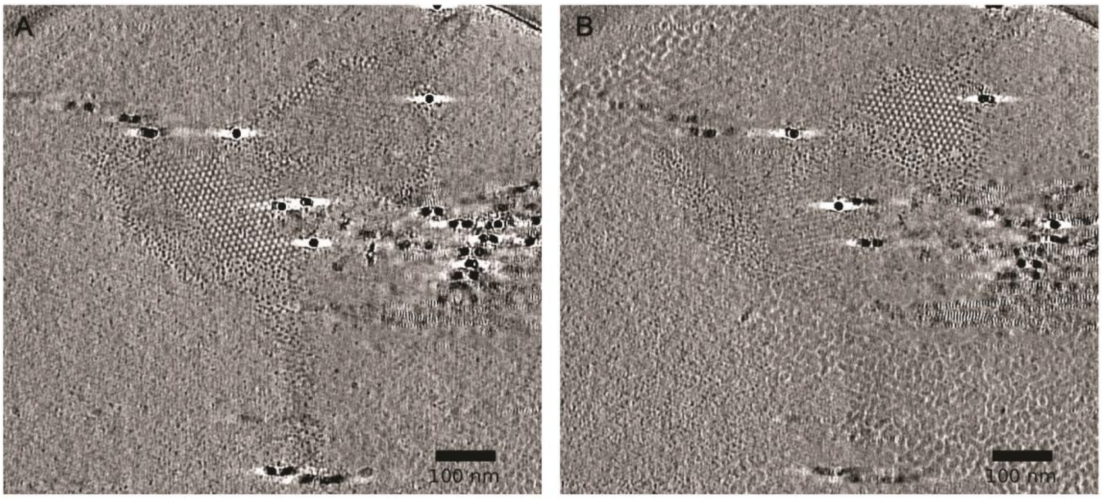

Figure R2 – *Honeycomb lattice in chimeric RSV/IAV particles.* Tomograms of a pleomorphic particle show extensive patches of glycoprotein arrays with hexagonal packing. Intriguingly, density underlying the array shows striped density – possibly the M-array. Slices are shown at different z values through the same particle (A,B).

- The finding that glycoproteins cluster in pairs is really interesting. However, in the absence of any subtomogram averaging this observation cannot be interpreted. Performing the averaging suggested by the authors in the discussion would greatly improve the significance of the manuscript.

We agree that sub-tomogram averaging of the glycoprotein doublets would be a highly desirable study, however as noted in our response to reviewer #1 we are not able to provide these data in a timely manner and are not keen to delay the publication of the present study unduly.

Other minor comments:

- Figure 1 does not have a panel D, but the legend describes it.

Panel D has been reinstated with accompanying description as requested by the reviewer.

- Page 3, "Tomographic reconstruction of filament sections reveal that they are well-ordered." should be "Tomographic reconstruction of filament sections reveal that virions are well-ordered."

Text has been amended.

Dear David,

Thanks for submitting your revised manuscript to The EMBO Journal. Your study has now been seen by two referees and their comments are provided below. As you can see from the comments, the referees appreciate the introduced changes and support publication here. They have a few minor comments (ref #3) to address. Regarding one of the comments raided by referee #2, the EMBO Journal doesn't allow not data not shown to be referenced in the manuscript.

When you submit the revised manuscript will you please also address the following concerns:

We need 3-5 keywords

Data Deposition should be relabeled as Data Availability

Callouts are missing for EV1 + EV2

The MOVIE: The movies play and include the legend within the file. The name of the movies should be corrected to 'Movie EV#'. The callout in text is fine. fine.

The figure legends should be moved to after the Reference list.

Please add the heading 'Expanded View Figure Legends' to the manuscript text.

Our publisher has also done their pre-publication check on your manuscript. When you log into the manuscript submission system you will see the "data edited ms" file. Please look at the word file and the comments regarding the figure legends and Data Availability section and respond to these.

We include a synopsis of the paper (see http://emboj.embopress.org/). Please provide me with a general summary statement and 3-5 bullet points that capture the key findings of the paper.

We also need a summary figure for the synopsis. The size should be 550 wide by [200-400] high (pixels). You can also use something from the figures if that is easier.

I think that should be it.

Let me know if you have any further questions

With best wishes

Karin

Karin Dumstrei, PhD
Senior Editor
The EMBO Journal

Guide For Authors: https://www.embopress.org/page/journal/14602075/authorguide

The revision must be submitted online within 90 days; please click on the link below to submit the revision online before 28th Feb 2022.

https://emboj.msubmit.net/cgi-bin/main.plex

Referee #1:

After the review all my doubts have been addressed. I congratulate the authors for this excellent work.

Referee #3:

The authors have addressed my concerns. However, the authors should include in the text their answers to the reviewers regarding filament diameters from U2-OS and A549 cells and the alternate fits of the M-protein dimers. Additionally, the images of the honeycomb lattice from chimeric RSV/IAV particles should be included as a supplementary figure. If the editorial policy of EMBO J allow it, the authors should also mention the hexagonal surface features in negative stain images of purified RSV virions.

2nd Revision - Editorial Decision 3rd Dec 2021

Dear David,

Thank you for submitting your revised manuscript to The EMBO Journal. I have now had a chance to look at the revised version and all looks good!

I am therefore very pleased to accept the manuscript for publication here.

Congratulation on a nice study

Best Karin

Karin Dumstrei, PhD
Senior Editor
The EMBO Journal

Please note that it is EMBO Journal policy for the transcript of the editorial process (containing referee reports and your response letter) to be published as an online supplement to each paper. If you do NOT want this, you will need to inform the Editorial Office via email immediately. More information is available here:
https://www.embopress.org/page/journal/14602075/authorguide#transparentprocess

Your manuscript will be processed for publication in the journal by EMBO Press. Manuscripts in the PDF and electronic editions of The EMBO Journal will be copy edited, and you will be provided with page proofs prior to publication. Please note that supplementary information is not included in the proofs.

Please note that you will be contacted by Wiley Author Services to complete licensing and payment information. The 'Page Charges Authorization Form' is available here: https://www.embopress.org/pb-assets/embo-site/tej_apc.pdf

Should you be planning a Press Release on your article, please get in contact with embojournal@wiley.com as early as possible, in order to coordinate publication and release dates.

If you have any questions, please do not hesitate to call or email the Editorial Office. Thank you for your contribution to The EMBO Journal.

** Click here to be directed to your login page: https://emboj.msubmit.net

EMBO PRESS

YOU MUST COMPLETE ALL CELLS WITH A PINK BACKGROUND ➔

PLEASE NOTE THAT THIS CHECKLIST WILL BE PUBLISHED ALONGSIDE YOUR PAPER

Corresponding Author Name: David Bhella
Journal Submitted to: EMBO Journal
Manuscript Number: EMBOJ-2021-109728

USEFUL LINKS FOR COMPLETING THIS FORM

http://www.antibodypedia.com
http://1degreebio.org
http://www.equator-network.org/reporting-guidelines/improving-bioscience-research-repor
http://grants.nih.gov/grants/olaw/olaw.htm
http://www.mrc.ac.uk/Ourresearch/Ethicsresearchguidance/Useofanimals/index.htm
http://ClinicalTrials.gov
http://www.consort-statement.org
http://www.consort-statement.org/checklists/view/32-consort/66-title
http://www.equator-network.org/reporting-guidelines/reporting-recommendations-for-tumo
http://datadryad.org
http://figshare.com
http://www.ncbi.nlm.nih.gov/gap
http://www.ebi.ac.uk/ega
http://biomodels.net/
http://biomodels.net/miriam/
http://jjj.biochem.sun.ac.za
https://osp.od.nih.gov/biosafety-biosecurity-and-emerging-biotechnology/
http://www.selectagents.gov/

Reporting Checklist For Life Sciences Articles (Rev. June 2017)

This checklist is used to ensure good reporting standards and to improve the reproducibility of published results. These guidelines are consistent with the Principles and Guidelines for Reporting Preclinical Research issued by the NIH in 2014. Please follow the journal's authorship guidelines in preparing your manuscript.

A- Figures
1. Data
The data shown in figures should satisfy the following conditions:

- ➔ the data were obtained and processed according to the field's best practice and are presented to reflect the results of the experiments in an accurate and unbiased manner.
- ➔ figure panels include only data points, measurements or observations that can be compared to each other in a scientifically meaningful way.
- ➔ graphs include clearly labeled error bars for independent experiments and sample sizes. Unless justified, error bars should not be shown for technical replicates.
- ➔ if n< 5, the individual data points from each experiment should be plotted and any statistical test employed should be justified
- ➔ Source Data should be included to report the data underlying graphs. Please follow the guidelines set out in the author ship guidelines on Data Presentation.

2. Captions
Each figure caption should contain the following information, for each panel where they are relevant:

- ➔ a specification of the experimental system investigated (eg cell line, species name).
- ➔ the assay(s) and method(s) used to carry out the reported observations and measurements
- ➔ an explicit mention of the biological and chemical entity(ies) that are being measured.
- ➔ an explicit mention of the biological and chemical entity(ies) that are altered/varied/perturbed in a controlled manner.

- ➔ the exact sample size (n) for each experimental group/condition, given as a number, not a range;
- ➔ a description of the sample collection allowing the reader to understand whether the samples represent technical or biological replicates (including how many animals, litters, cultures, etc.).
- ➔ a statement of how many times the experiment shown was independently replicated in the laboratory.
- ➔ definitions of statistical methods and measures:
  - common tests, such as t-test (please specify whether paired vs. unpaired), simple $\chi 2$ tests, Wilcoxon and Mann-Whitney tests, can be unambiguously identified by name only, but more complex techniques should be described in the methods section;
  - are tests one-sided or two-sided?
  - are there adjustments for multiple comparisons?
  - exact statistical test results, e.g., P values = x but not P values < x;
  - definition of 'center values' as median or average;
  - definition of error bars as s.d. or s.e.m.

Any descriptions too long for the figure legend should be included in the methods section and/or with the source data.

In the pink boxes below, please ensure that the answers to the following questions are reported in the manuscript itself. Every question should be answered. If the question is not relevant to your research, please write NA (non applicable). We encourage you to include a specific subsection in the methods section for statistics, reagents, animal models and human subjects.

B- Statistics and general methods

Please fill out these boxes ➔ (Do not worry if you cannot see all your text once you press return)

1.a. How was the sample size chosen to ensure adequate power to detect a pre-specified effect size?	NA
1.b. For animal studies, include a statement about sample size estimate even if no statistical methods were used.	NA
2. Describe inclusion/exclusion criteria if samples or animals were excluded from the analysis. Were the criteria pre-established?	NA
3. Were any steps taken to minimize the effects of subjective bias when allocating animals/samples to treatment (e.g. randomization procedure)? If yes, please describe.	NA
For animal studies, include a statement about randomization even if no randomization was used.	NA
4.a. Were any steps taken to minimize the effects of subjective bias during group allocation or/and when assessing results (e.g. blinding of the investigator)? If yes please describe.	No
4.b. For animal studies, include a statement about blinding even if no blinding was done	NA
5. For every figure, are statistical tests justified as appropriate?	YES
Do the data meet the assumptions of the tests (e.g., normal distribution)? Describe any methods used to assess it.	YES
Is there an estimate of variation within each group of data?	YES

Is the variance similar between the groups that are being statistically compared?	No - in the assessment of filament diameters produced by two cell-lines given in the response to reviewers comments, the variance was calculated to be significantly different.

C- Reagents

6. To show that antibodies were profiled for use in the system under study (assay and species), provide a citation, catalog number and/or clone number, supplementary information or reference to an antibody validation profile. e.g., Antibodypedia (see link list at top right), 1DegreeBio (see link list at top right).	NA
7. Identify the source of cell lines and report if they were recently authenticated (e.g., by STR profiling) and tested for mycoplasma contamination.	All cell-lines are routinely tested for mycoplasma contamination

* for all hyperlinks, please see the table at the top right of the document

D- Animal Models

8. Report species, strain, gender, age of animals and genetic modification status where applicable. Please detail housing and husbandry conditions and the source of animals.	NA
9. For experiments involving live vertebrates, include a statement of compliance with ethical regulations and identify the committee(s) approving the experiments.	NA
10. We recommend consulting the ARRIVE guidelines (see link list at top right) (PLoS Biol. 8(6), e1000412, 2010) to ensure that other relevant aspects of animal studies are adequately reported. See author guidelines, under 'Reporting Guidelines'. See also: NIH (see link list at top right) and MRC (see link list at top right) recommendations. Please confirm compliance.	NA

E- Human Subjects

11. Identify the committee(s) approving the study protocol.	NA
12. Include a statement confirming that informed consent was obtained from all subjects and that the experiments conformed to the principles set out in the WMA Declaration of Helsinki and the Department of Health and Human Services Belmont Report.	NA
13. For publication of patient photos, include a statement confirming that consent to publish was obtained.	NA
14. Report any restrictions on the availability (and/or on the use) of human data or samples.	NA
15. Report the clinical trial registration number (at ClinicalTrials.gov or equivalent), where applicable.	NA
16. For phase II and III randomized controlled trials, please refer to the CONSORT flow diagram (see link list at top right) and submit the CONSORT checklist (see link list at top right) with your submission. See author guidelines, under 'Reporting Guidelines'. Please confirm you have submitted this list.	NA
17. For tumor marker prognostic studies, we recommend that you follow the REMARK reporting guidelines (see link list at top right). See author guidelines, under 'Reporting Guidelines'. Please confirm you have followed these guidelines.	NA

F- Data Accessibility

18: Provide a "Data Availability" section at the end of the Materials & Methods, listing the accession codes for data generated in this study and deposited in a public database (e.g. RNA-Seq data: Gene Expression Omnibus GSE39462, Proteomics data: PRIDE PXD000208 etc.) Please refer to our author guidelines for 'Data Deposition'.<br><br>Data deposition in a public repository is mandatory for:<br>a. Protein, DNA and RNA sequences<br>b. Macromolecular structures<br>c. Crystallographic data for small molecules<br>d. Functional genomics data<br>e. Proteomics and molecular interactions	Data Deposition statement is provided.
19. Deposition is strongly recommended for any datasets that are central and integral to the study; please consider the journal's data policy. If no structured public repository exists for a given data type, we encourage the provision of datasets in the manuscript as a Supplementary Document (see author guidelines under 'Expanded View' or in unstructured repositories such as Dryad (see link list at top right) or Figshare (see link list at top right).	Following discussion with the editor, we shall not in this case deposit the raw micrograph movies as we are still analysing the data and hope to determine further structures. A representative tomogram and the sub-tomogram average is deposited in the EM databank.
20. Access to human clinical and genomic datasets should be provided with as few restrictions as possible while respecting ethical obligations to the patients and relevant medical and legal issues. If practically possible and compatible with the individual consent agreement used in the study, such data should be deposited in one of the major public access-controlled repositories such as dbGAP (see link list at top right) or EGA (see link list at top right).	NA
21. Computational models that are central and integral to a study should be shared without restrictions and provided in a machine-readable form. The relevant accession numbers or links should be provided. When possible, standardized format (SBML, CellML) should be used instead of scripts (e.g. MATLAB). Authors are strongly encouraged to follow the MIRIAM guidelines (see link list at top right) and deposit their model in a public database such as Biomodels (see link list at top right) or JWS Online (see link list at top right). If computer source code is provided with the paper, it should be deposited in a public repository or included in supplementary information.	NA

G- Dual use research of concern

22. Could your study fall under dual use research restrictions? Please check biosecurity documents (see link list at top right) and list of select agents and toxins (APHIS/CDC) (see link list at top right). According to our biosecurity guidelines, provide a statement only if it could.	No
